# Weight, socio-demographics, and health behaviour related correlates of academic performance in first year university students

**DOI:** 10.1186/1475-2891-12-162

**Published:** 2013-12-17

**Authors:** Tom Deliens, Peter Clarys, Ilse De Bourdeaudhuij, Benedicte Deforche

**Affiliations:** 1 Department of Human Biometry and Biomechanics, Vrije Universiteit Brussel, Brussels, Belgium; 2 Department of Movement and Sports Sciences, Ghent University, Ghent, Belgium

**Keywords:** Weight, Health behaviour, Correlates, Academic performance, First year university students

## Abstract

**Background:**

This study aimed to examine differences in socio-demographics and health behaviour between Belgian first year university students who attended all final course exams and those who did not. Secondly, this study aimed to identify weight and health behaviour related correlates of academic performance in those students who attended all course exams.

**Methods:**

Anthropometrics of 101 first year university students were measured at both the beginning of the first (T1) and second (T2) semester of the academic year. An on-line health behaviour questionnaire was filled out at T2. As a measure of academic performance student end-of-year Grade Point Averages (GPA) were obtained from the university’s registration office. Independent samples *t*-tests and *chi*^*2*^-tests were executed to compare students who attended all course exams during the first year of university and students who did not carry through. Uni- and multivariate linear regression analyses were conducted to identify correlates of academic performance in students who attended all course exams during the first year of university.

**Results:**

Students who did not attend all course exams were predominantly male, showed higher increases in waist circumference during the first semester and consumed more French fries than those who attended all final course exams. Being male, lower secondary school grades, increases in weight, Body Mass Index and waist circumference over the first semester, more gaming on weekdays, being on a diet, eating at the student restaurant more frequently, higher soda and French fries consumption, and higher frequency of alcohol use predicted lower GPA’s in first year university students. When controlled for each other, being on a diet and higher frequency of alcohol use remained significant in the multivariate regression model, with frequency of alcohol use being the strongest correlate of GPA.

**Conclusions:**

This study, conducted in Belgian first year university students, showed that academic performance is associated with a wide range of weight and health related behaviours. Future studies should investigate whether interventions aiming at promoting healthy behaviours among students could also have a positive impact on academic performance.

## Background

Higher academic performance during the years at university is highly related to career success [1]. Moreover, academic performance influences future educational attainment and income, which, in turn, affect health and quality of life [1,2]. Therefore, determining factors related with academic grades is important to both universities [3] and their undergraduates.

Some authors indicated that secondary school average was the strongest positive predictor of academic performance in both male and female university students [4-6]. Furthermore, different studies agreed that female university students tended to have greater academic success [3,6,7]. In a study of Singleton et al. [5] in US college students parental education as well as ethnicity (being Caucasian) correlated positively with academic performance. Although students living in a student residence are assumed to experience less parental control, which might influence academic efforts and achievement negatively, no previous studies investigated the role of residency (on or off campus) on academic performance in university students.

It has been shown in literature that physical activity participation as well as more healthy diets (i.e. diets low in saturated fats and refined sugars) are beneficial to cognition [8,9], suggesting that health behaviours may affect cognitive performances. University, and especially the freshman year, is a period in which students are often subject to several health behaviour related and lifestyle changes [10-12]. Hence, Grade Point Averages (GPA), which is often used as a measure for academic performance, might be affected by such health behaviour changes during the first year at university. Although studies in secondary school students have shown associations between several health behaviours (physical activity, sedentary lifestyle and diet) and academic performance [13-15], literature relating health behaviour to academic performance in university students is scarce and limited to US studies. A recent study revealed that university students who engaged in strength exercise more frequently had significantly higher GPA’s [16]. Regarding the relation between sedentary behaviour and academic performance, playing videogames was found to be negatively related to GPA [17]. No relationships between other sedentary activities (e.g. TV watching, reading and studying, computer activities) and academic performance in university students have been examined so far. In a study in first year university students, Trockel et al. [3] only found a positive relation with frequency of eating breakfast, whereas no other diet-related variables (e.g. fruit and vegetable intake) were significantly related to academic performance. Several authors found a negative correlation between alcohol consumption and academic performance [5,6,18]. Smoking was also negatively related to GPA [6]. Furthermore, the review study of Curcio et al. [19], conducted in all student age categories, showed a clear negative relation between sleep quality and quantity, and student learning capacity and academic performance. However, other work did not find a significant correlation between total sleep time and academic achievement [20]. Finally, the review by Richardson et al. [21] indicated that stress was negatively related to academic achievement. To the best of our knowledge no studies have assessed the association between dieting status or body composition and academic performance in university students.

In summary, only a limited number of studies (mostly conducted in the US) examined the relationship between end-of-year performance and certain health behaviours (e.g. physical activity, dietary behaviour) in university students, while relations with other health behaviours (e.g. dieting status) and body composition have not been studied yet. Due to socio-cultural differences we cannot extrapolate these US findings to European university students. Hence, next to what is known from previous US studies, this manuscript should provide complementary European evidence on how weight, socio-demographics and health related behaviours relate to academic performance in this population. Therefore, the first purpose of this study was to examine differences in socio-demographics and health behaviour between Belgian (European) first year university students who attended all course exams and received a GPA (and either passed or failed) and those who quit university or did not carry through all exams and therefore did not receive a GPA. The second purpose of this study was to investigate a wide range of weight and health behaviour related correlates of academic performance in those students who attended all course exams during the first year of university and therefore received a GPA.

## Methods

### Participants

Convenience sampling (via e-mail and face-to-face recruitment) was used to recruit Belgian first year university students at the Vrije Universiteit Brussel. Hundred and one participants were prepared to participate and completed all anthropometric measurements and a health behaviour questionnaire. Since self-reported data of all first year university students (=924) were obtained during enrolment (by the university’s registration office), we were able to verify the representativeness of the sample. The study sample (21.8 ± 2.8 kg/m^2^) was representative in comparison to all freshmen in Body Mass Index (BMI) (21.4 ± 2.8 kg/m^2^, *t* = -1.1; *p* = 0.262), but was slightly younger (18.0 ± 0.7 yrs; 18.5 ± 1.1 yrs, *t* = 5.6; *p* < 0.001) and consisted of a higher proportion of female participants (67.3% versus 56.6% females, *chi*^*2*^ = 4.3; *p* = 0.039).

### Procedure

Anthropometric measurements were conducted at both the beginning of the first and second semester of the academic year (October/November 2011 (T1) and February/March 2012 (T2)). The on-line questionnaire was only filled out at T2. All anthropometric tests were conducted in the anthropometrics laboratory at the Faculty of Physical Education and Physiotherapy, whereas the on-line questionnaire could be filled out at home. To minimize possible effects due to sudden short-term lifestyle changes after holiday periods, measurements were conducted after two weeks of academic activities. At both time points students received an incentive (a lunch voucher). An informed consent was signed by all participants before the start of the study. The study protocol was approved by the medical ethical committee of the university hospital. More detailed methodological information can be found elsewhere [12].

### Measurements

#### Anthropometrics

At both time points, all students were measured with bare feet, wearing light sports clothing (shorts and t-shirt). They were asked to void their bladder before starting the measurements as well as to remove all jewellery. Weight (TANITA BC-418 MA Body Composition Analyzer) and height (wall-fixed stadiometer) were measured to calculate BMI. Fat% (TANITA for bio-electrical impedance analysis) and waist circumference (WC) (measured with Rosscraft Anthrotape on the narrowest part of the waist) were measured to analyse body composition and fat distribution. All measurements, except those using TANITA, were conducted twice and an additional third time when tolerance limits were exceeded. Subsequently, mean values were calculated from the two nearest values.

### Health behaviour questionnaire

At T2 all participants were also asked to complete a self-reported on-line questionnaire assessing demographics, socio-economic status, smoking, dieting status, physical activity, sedentary behaviour, dietary habits, sleeping habits and mental stress. These behaviours were assessed as literature demonstrated their potential contribution to academic performance in secondary school or college/university students [3,15,18,22]. The questionnaire consisted of questions derived from existing questionnaires (Project EAT-II Survey for Young Adults [23], Health Behaviour in School-aged Children (HBSC) [24], Health and Behaviour Survey (HBS) [25], Flemish Physical Activity Questionnaire (FPAQ) [26], Perceived Stress Scale (PSS) [27]). All items included in the questionnaire as well as reference to the original questionnaire can be found in Table 1.

**Table 1:** Descriptive statistics of possible influencing factors of GPA in first year university students (%, Mean ± SD, n = 101), subdivided into students who passed (n = 52), failed (n = 22) or did not attend all final course exams (n = 27)

**Measures**	**All**	**Passed**	**Failed**	**Did not attend all course exams n = 27**
	**n = 74**	**n = 52**	**n = 22**	
***GPA (%)***	64.3 ± 9.2	68.3 ± 6.9	54.7 ± 6.2	/
***Demographics***				
Gender (% of females)	77.0	82.7	63.6	40.7
Age (yrs)	18.0 ± 0.6	17.9 ± 0.5	18.1 ± 0.8	18.3 ± 0.9
Ethnicity (% of students of which one of the parents is from foreign origin)	20.9	23.3	15.4	20.0
Residency (% living in student residence)	47.3	48.1	45.5	25.9
GPA in the last year of secondary school (%)	68.6 ± 7.5	70.0 ± 6.9	65.0 ± 8.0	63.6 ± 6.3
***Socio-Economic Status (SES)***^***c***^				
Education father (% diploma higher education)	57.2	48.3	77.0	46.7
Education mother (% diploma higher education)	69.0	70.0	66.6	60.0
***Smoking (% non-smokers)***	95.9	96.2	95.5	96.2
***Dieting status (% dieters)***^***b***^	11.0	9.8	13.6	11.5
***Anthropometrics***				
Initial weight (kg)	61.8 ± 9.3	61.0 ± 8.0	63.5 ± 12.0	66.7 ± 14.0
Initial BMI (kg/m^2^)	21.7 ± 2.7	21.5 ± 2.5	22.0 ± 3.1	22.1 ± 3.3
Initial fat% (%)	22.5 ± 7.1	22.8 ± 7.4	21.9 ± 6.7	19.2 ± 6.5
Initial WC (cm)	22.5 ± 7.1	22.8 ± 7.4	21.9 ± 6.7	19.2 ± 6.5
Weight change (kg)	0.7 ± 2.0	0.4 ± 1.9	1.6 ± 1.8	1.6 ± 2.2
BMI change (kg/m^2^)	0.3 ± 0.8	0.1 ± 0.8	0.5 ± 0.7	0.4 ± 0.8
Fat% change (%)	1.0 ± 2.4	0.7 ± 2.5	1.5 ± 2.0	0.5 ± 2.8
WC change (cm)	-0.0 ± 2.3	-0.5 ± 2.3	1.0 ± 2.1	1.2 ± 2.2
***Physical activity***^***d***^				
Active transportation (walking and cycling) (min/week)	179.7 ± 123.5	175.7 ± 119.5	189.5 ± 135.3	193.5 ± 118.4
Sport participation (min/week)	146.1 ± 180.1	152.7 ± 180.4	130.7 ± 182.9	117.4 ± 161.0
Total physical activity (min/week)	324.8 ± 211.7	326.8 ± 220.3	320.2 ± 195.4	310.8 ± 222.8
***Sedentary behaviour***^***a***^				
TV/DVD watching on weekdays (hours/day)	1.2 ± 0.8	1.2 ± 0.8	1.1 ± 0.8	1.2 ± 0.8
TV/DVD watching on weekend days (hours/day)	2.1 ± 1.1	2.1 ± 1.2	1.9 ± 1.0	2.2 ± 1.3
Reading and studying on weekdays (hours/day)	1.8 ± 1.1	1.8 ± 1.2	1.9 ± 0.8	1.9 ± 1.1
Reading and studying on weekend days (hours/day)	2.9 ± 1.5	2.8 ± 1.6	3.1 ± 1.5	2.8 ± 1.5
Computer activities on week days (hours/day)	1.7 ± 1.3	1.7 ± 1.4	1.7 ± 1.2	1.9 ± 1.2
Computer activities on weekend days (hours/day)	1.9 ± 1.2	1.8 ± 1.3	2.1 ± 1.0	2.3 ± 1.5
Video games on weekdays (hours/day)	0.2 ± 0.6	0.1 ± 0.4	0.4 ± 0.9	0.3 ± 1.0
Video games on weekend days (hours/day)	0.4 ± 1.0	0.3 ± 0.9	0.5 ± 1.2	0.6 ± 1.1
***Eating habits***				
Eating breakfast (#/week)^a^	5.7 ± 2.2	5.5 2.3	6.0 ± 2.0	5.8 ± 2.3
Eating lunch (#/week)^a^	6.6 ± 1.2	6.5 ± 1.3	6.6 ± 1.0	6.7 ± 0.9
Eating dinner (#/week)^a^	6.7 ± 0.9	6.7 ± 0.7	6.6 ± 1.3	6.8 ± 0.5
Eating at home with parents (#/week)^a^	3.8 ± 2.1	3.6 ± 2.1	4.1 ± 2.1	4.6 ± 2.1
Eating at student restaurant (#/week)^a^	1.2 ± 1.5	1.0 ± 1.1	1.8 ± 2.1	1.8 ± 1.9
Eating at fast food restaurant (#/week)^a^	0.3 ± 0.4	0.3 ± 0.4	0.3 ± 0.3	0.4 ± 0.3
Eating at other kind of restaurant (#/week)^a^	0.3 ± 0.3	0.3 ± 0.3	0.3 ± 0.3	0.4 ± 0.3
Eating at a friend’s place (#/week)^a^	0.4 ± 0.5	0.4 ± 0.4	0.4 ± 0.6	0.5 ± 0.5
Fruit consumption (#/day)^b^	1.0 ± 1.0	1.0 ± 1.1	0.9 ± 0.6	1.0 ± 1.1
Vegetable consumption (#/day)^b^	1.2 ± 0.7	1.2 ± 0.7	1.2 ± 0.6	1.3 ± 1.0
Soda consumption (#/day)^b^	0.8 ± 1.1	0.6 ± 0.9	1.2 ± 1.3	1.2 ± 1.3
French fries consumption (#/week)^b^	0.1 ± 0.1	0.1 ± 0.1	0.1 ± 0.1	0.1 ± 0.1
Fast food consumption (#/week)^b^	0.7 ± 0.9	0.7 ± 0.9	0.8 ± 0.9	0.9 ± 0.9
***Alcohol***				
Frequency of alcohol use (#/week)^b^	0.8 ± 1.5	0.6 ± 1.0	1.3 ± 2.4	0.8 ± 1.3
Frequency of alcohol consumptions (# on drinking days)^c^	2.7 ± 2.0	2.6 ± 1.9	2.9 ± 2.2	3.1 ± 3.0
***Sleeping habits***^***c***^				
Hours of sleep on weekdays (hours/day)	7.8 ± 1.0	7.8 ± 1.0	7.9 ± 1.1	7.6 ± 0.9
Hours of sleep on weekend days (hours/day)	9.4 ± 1.2	9.4 ± 1.2	9.3 ± 1.3	9.2 ± 1.2
***Stress***				
Mental stress (PSS score*)^e^	13.6 ± 5.9	13.5 ± 6.0	13.8 ± 5.8	14.4 ± 6.5

*Higher perceived stress scores (max = 40) indicate higher stress levels. Mean PSS score in age 18–29 was 14.2 ± 6.2 [27].

^a^Project EAT-II Survey for Young Adults [23].

^b^HBSC [24].

^c^HBS [25].

^d^FPAQ [26].

^e^PSS [27].

### GPA

Finally, after the end of the academic year GPA’s (in%) of students who attended all course exams were obtained from the university’s registration office as a measure of academic performance.

### Data analyses

SPSS Statistics 20 was used for data analyses. Independent samples *t*-tests and *chi*^*2*^-tests were executed to compare students who attended all course exams during the first year of university and students who did not carry through. A multiple linear regression analysis was conducted to identify correlates of academic performance in students who attended all course exams during the first year of university. Before running the multiple regression analysis, GPA was regressed onto each demographic variable and health behaviour measured at T2, all initial anthropometric variables at T1, as well as anthropometric changes over time (T2-T1). After checking for multicollinearity (*r* > 0.5) only significant correlates of GPA were included into the multiple regression analysis. *p*-Values < 0.05 were considered as statistically significant, whereas *p*-values < 0.1 were considered as trends towards significance.

## Results

### Comparison of characteristics and health related behaviour between students who attended all course exams during the first year of university and those who did not carry through

Of the 101 participants, 51.5% passed (n = 52) and 21.8% failed (n = 22) their final exams, whereas 26.7% either quit university, or did not attend all course exams and therefore did not receive a final GPA. Before examining correlates of GPA, a comparison between students who attended all final course exams (n = 74) and those who did not (n = 27) was made to assess possible differences in student socio-demographics and health related behaviours (shown in Table 1). Students who attended all course exams and students who did not showed a similar BMI at baseline (21.7 ± 2.7 kg/m^2^; 21.1 ± 3.3 kg/m^2^; *t* = 0.6, *p* = 0.535). Mean age (18.0 ± 0.6 yrs; 18.3 ± 0.9 yrs; *t* = 1.7; *p* = 0.101) was also similar, but the majority of students who attended all course exams were female (77.0%), whereas the majority of students who did not carry through were male (59.3%) (*chi*^*2*^ = 11.8; *p* = 0.001). Average GPA in the last year of secondary school was higher in students who carried through all course exams during the first year of university (68.6 ± 7.5%; n = 42) in comparison to those who did not (63.6 ± 6.3%; n = 14; *t* = -2.2; *p* = 0.030). Students who did not attend all course exams showed a significantly higher increase in WC (1.2 ± 2.2 vs. -0.0 ± 2.3; *t* = 2.4; *p* = 0.020) during the first semester. Regarding eating habits, only French fries consumption was slightly but significantly higher in students who did not attend all course exams (0.14 ± 0.11 #/week; 0.09 ± 0.08 #/week; *t* = 2.2; *p* = 0.028). No significant differences were found in physical activity, sedentary behaviour, alcohol consumption, sleeping habits and stress levels between both groups.

### Correlates of GPA

Descriptive statistics of all items tested as possible correlates of first year GPA at university are shown in Table 1.

Results of the univariate regression analyses (see Table 2) indicated that female students and students who had higher GPA’s in the last year of secondary school showed higher GPA’s in their first year at university. Weight gain was negatively correlated with academic performance, i.e. the more students had gained weight during the first semester at university, the lower their first year GPA’s. Similar negative correlations were found for increase in BMI and WC. Students who engaged more in gaming activities on weekdays seemed to score lower grades as well. Regarding eating behaviour, lower academic performance was found when students reported being on a diet, eating more frequently at the student restaurant, consumed more soda or French fries, or reported more weekly alcohol use. Computer activities on weekend days showed a negative trend (*p* < 0.1) towards significance.

**Table 2:** **Correlates of GPA in first year university students (t-values, β-values and Adjusted R**^**2**^**, n = 74)**

**Correlates**	***t***	***β***	***Adj R***^***2***^
***Univariate***			
Gender (0 = male; 1 = female)	3.1**	0.344	0.106
GPA last year secondary school (n = 42)	2.8**	0.401	0.140
Weight change	-3.1**	-0.340	0.103
BMI change	-2.4*	-0.273	0.062
WC change	-3.7***	-0.396	0.145
Gaming on weekdays	-2.2*	-0.251	0.050
Computer activities on weekend days	-1.7^	-0.195	0.024
Dieting status (0 = not currently dieting; 1 = currently dieting)	-2.4*	-0.273	0.062
Eating at student restaurant	-2.3*	-0.262	0.055
Soda consumption	-2.7**	-0.307	0.082
French fries consumption	-2.9**	-0.328	0.095
Frequency of alcohol use	-2.3*	-0.262	0.056
***Multivariate***			
Gender (0 = male; 1 = female)	1.3	0.164	
WC change	-1.0	-0.122	
Gaming on weekdays	-0.8	-0.096	
Dieting status	-2.1*	-0.212	
Eating at student restaurant	-1.9^	-0.224	
Soda consumption	-2.0^	-0.227	
French fries consumption	-0.3	-0.041	
Frequency of alcohol use	-2.1*	-0.218	
			Adj R^2^ = 0.30

*α* = 0.05, *p < 0.05, **p < 0.01, ***p < 0.001, ^ = trend towards significance (*p* < 0.1).

Before running the multivariate model we checked for multicollinearity and excluded weight change and BMI change which both showed *r* > 0.5 with WC change. As WC change showed the strongest correlation with the dependent variable, this variable was included in the multivariate model. In addition, we decided to exclude secondary school GPA as well because of the limited sample size (n = 42). After inclusion of all remaining significant univariate variables, 30% of total variance in academic performance was explained by the multivariate regression model. In this multivariate model, only dieting status and frequency of alcohol use remained significant. Eating at the student restaurant and soda consumption showed negative trends towards significance (*p* < 0.1).

## Discussion

The purpose of this study was to examine differences in socio-demographics and health behaviour between Belgian (European) first year university students who attended all final course exams and those who did not, as well as to identify weight and health behaviour related correlates of academic performance in those students who attended all course exams. Results indicated that female students showed higher GPA’s in the first year at university. Furthermore, increases in (over) weight over the first semester, sedentary behaviours, unhealthy eating and drinking behaviours negatively predicted academic performance.

In agreement with findings of previous US studies [3,6,7,21], female college or university students tended to have higher grades than male students. This could be partly explained by the fact that women more often show motivation, discipline and time management skills that are important to perform well in higher education [28,29]. Moreover, the study of Sheard et al. [29] revealed that female university students also reported a significantly higher mean score on commitment compared to male students, whilst commitment was the most important positive correlate of academic grades. The latter increases the likelihood of female university students achieving higher grades than their male counterparts [28]. Furthermore, we observed the same trend when comparing students who attended all course exams with those who did not, i.e. the majority of students who did not attend all course exams during the first year at university were male. Similar to previous findings in the literature [4-6] GPA in the last year of secondary school predicted first year university GPA positively.

Increases in weight, BMI and WC over the first semester period were related to lower academic performance. Additionally, students who did not attend all course exams during the first year of university showed a significantly higher increase in WC during the first semester than those who attended all final course exams. Since no literature on this matter was available, no comparison could be made. However, these results indicated that increases in weight, BMI and WC may have affected students’ cognitive performances. It might also be that students who lacked discipline to maintain weight were equally not disciplined to perform well academically. Furthermore, it has been shown that medically defined overweight status is negatively associated with academic achievement [30,31].

Similar to other studies conducted in US university students [3,4], our results indicated that physical activity was not related to academic performance. This finding is in contrast with the review study of Singh et al. [22] relating physical activity with academic performance in children and adolescents. This latter study found strong evidence of a significant positive relationship between physical activity and academic performance. According to Hillman et al. [8] regular participation in physical activity is linked to enhancement of brain function and cognition, and thus positively influencing academic performance. In Singh et al. [22], several hypothesized physiological mechanisms (e.g. increased blood and oxygen flow to the brain and increased levels of endocrine processes) explaining the beneficial effect of exercise on cognition are given. In addition, regular participation in sport activities may improve students’ classroom behaviour and concentration during the lessons [22]. Therefore, we could expect that students who were more engaged in physical activities would perform better academically. However, on the other hand, when spending more time exercising, students possibly tended to spend less time on their academic work, so this might have offset the beneficial effects of physical activity on GPA at university.

Regarding sedentary behaviour, gaming on weekdays negatively predicted GPA. In addition, computer activities on weekend days showed a trend towards significance (*p* < 0.1). This suggests a negative influence of ‘screen activities’ on grade points in university students. Earlier research showed that the amount of time a university student spends playing video games is negatively related with GPA [17]. Similar to physical activity, this negative relationship might be explained by the fact that university students who spent more time playing videogames tended to spend less time studying. However, in this study, the amount of reading (incl. leisure time reading) and studying during the whole week did not correlate significantly with GPA. In contrast to the literature [19], the amount of sleep was not associated with academic performance.

Students who were on a diet, ate more in the student restaurant, consumed more (sugar containing or diet) sodas or ate French fries more frequently tended to have lower GPA’s. Similar results have been found in adolescents showing that unhealthy food choices (including fast foods) were negatively related, whereas fruit and vegetable consumption was positively related to secondary school end-of-year achievement [13,15]. In addition, Florence et al. [32] concluded that 10 year old students with decreased overall diet quality were more likely to perform poorly on an assessment used as a measure for academic performance. Moreover, dietary adequacy, variety, fruit and vegetable consumption and dietary fat intake were demonstrated as being important to academic performance [32]. Concerning the association between GPA and eating at the student restaurant, it could be that university students tend to make more unhealthy choices (e.g. more fatty foods and less fruit and vegetables) when eating in the campus restaurant. When controlled for other significant variables, being on a diet remained significant in the multivariate model. No previous correlational studies have assessed dieting status so far. These results suggest that unhealthy eating behaviours are related to lower academic performance.

Finally, even when controlled for other significant variables, frequency of alcohol use was associated with academic performance. The more frequent students drank alcohol, the lower their academic grades at the end of their first year at university. The latter confirmed the findings of Singleton et al. [5] showing a similar negative correlation between alcohol consumption and GPA in US university students. Furthermore, in a Belgian study, a 25% excess risk of failing at university was found in first year students who met alcohol dependence criteria [18].

According to our results many weight and health-related behaviours were associated with university students’ academic performance, with dieting status and alcohol use emerging as the strongest correlates of academic performance, once controlled for other significant variables. However, we should be careful interpreting these results. In a review and meta-analysis of Richardson et al. [21], several psychological correlates of university students’ academic performance were reported. Self-esteem, academic intrinsic motivation, and academic and performance self-efficacy were positively associated with GPA [21]. Goal commitment and social support showed a positive relation with GPA, whereas measures of psychological health (general and academic stress) were negatively correlated with academic achievement [21]. In addition, Chambel et al. [33] indicated that higher levels of academic satisfaction [33] influence student performance positively. Taking this into account, underlying psychological/motivational mechanisms might be mediating or confounding these relationships. Typically, a mediator is a third variable affecting the relation between two other variables [34]. E.g. previous research of Kristjansson et al. [14,15] in secondary school adolescents indicated a weak mediating role of both self-esteem and school contentment (satisfaction) on the relation between health behaviours and academic grades. A confounder, on the other hand, causes both the independent and the dependent variable [34]. It might also be that students who were more disciplined concerning their health behaviour, showed the same discipline regarding academic performance. E.g. lack of self-discipline might cause students to eat more unhealthy foods, and at the same time, it might cause these students to perform worse academically. A study of Duckworth et al. [35] showed that self-discipline accounted for more than twice as much variance as IQ in final grades in adolescents. Therefore, future studies should investigate possible mediation and/or confounder effects of psychological/motivational aspects on the relation between health behaviour and academic performance in university students.

A first strength of this study is that we were able to regress academic performance onto objectively measured first semester changes in weight, BMI, fat% and WC. Secondly, in contrast to other studies, we used objective GPA’s (obtained from official university records) instead of self-reports. However, this study has some limitations as well. Firstly, it should be mentioned that no information was collected to verify whether those who did not carry through did so for academic reasons, or reasons related to health. Therefore, it might be that students who did not attend all course exams did not automatically lack self-discipline or academic competences, but were forced to miss out on exams due to health related reasons. Secondly, since our health-related questionnaire was only completed at follow-up, we were not able to regress academic performance onto changes in physical activity, dietary habits, and other health behaviours over time. Thirdly, because of the limited sample size (n = 42) we chose to exclude secondary school GPA from the t-tests as well as from the multivariate regression model to ensure sufficient degrees of freedom. Subsequently, we did not control our multivariate regression model for secondary school GPA. Fourthly, we have to be aware that the use of objective measures of physical activity (e.g. accelerometers) and food frequency questionnaires or food diaries would have given us more accurate and detailed information on physical activity and dietary behaviour. Finally, due to a relatively small sample size with a slightly different mean age and gender distribution in comparison to all freshmen, we have to be cautious with generalizing these results to the entire student population.

## Conclusions

This is the first European study showing associations between a wide range of weight and health related behaviours and academic performance in first year university students. Students who did not attend all course exams were predominantly male, showed higher increases in WC during the first semester and consumed more French fries than those who carried through and attended all final course exams. Furthermore, gender, secondary school GPA, changes in weight, BMI and WC, gaming, dieting status, eating at the student restaurant, soda and French fries consumption, and alcohol consumption during the academic year were associated with end-of-year GPA in first year university students who attended all course exams. Future intervention studies aiming to improve students’ academic performances should pay special attention to male students with lower secondary school grades showing increases in weight, BMI and WC over the first semester, higher levels of sedentary behaviour and unhealthy eating and drinking habits. However, it remains to be investigated whether interventions aiming at promoting healthy behaviours among university students could indeed have a positive impact on academic performance.

## Abbreviations

BMI: Body mass index; FPAQ: Flemish physical activity questionnaire; GPA: Grade point average; HBS: Health and behaviour survey; HBSC: Health behaviour in school-aged children; PSS: Perceived stress scale; SES: Socio-economic status; SPSS: Statistical package for the social sciences; US: United states; WC: Waist circumference.

## Competing interests

The authors declare that they have no competing interest.

## Authors’ contributions

TD participated in the design of the study, collected all data, performed the data analyses and drafted the manuscript. IDB participated in the design of the study and revised the manuscript critically. BD and PC participated in the design of the study, contributed to the interpretation of data and revised the manuscript critically. All authors read and approved the final manuscript.

## References

[B1] Tan DL Grades as predictors of college and career success: the case of a health-related institution J Coll Admiss 1991 12 12 15

[B2] Ross CE Wu CL The Links between Education and Health Am Sociol Rev 1995 12 719 745

[B3] Trockel MT Barnes MD Egget DL Health-related variables and academic performance among first-year college students: implications for sleep and other behaviors J Am Coll Health 2000 12 125 131 11125640 10.1080/07448480009596294

[B4] Ruthig JC Marrone S Hladkyj S Robinson-Epp N Changes in college student health: implications for academic performance J Coll Stud Dev 2011 12 307 320

[B5] Singleton RA Collegiate alcohol consumption and academic performance J Stud Alcohol Drugs 2007 12 548 555 17568960 10.15288/jsad.2007.68.548

[B6] DeBerard MS Spielmans GI Julka DL Predictors of academic achievement and retention among college freshmen: a longitudinal study Coll Stud J 2004 12 66 80

[B7] Vaez M Laflamme L Experienced stress, psychological symptoms, self-rated health and academic achievement: a longitudinal study of Swedish university students Social Behavior and Personality 2008 12 183 195

[B8] Hillman CH Erickson KI Kramer AF Be smart, exercise your heart: exercise effects on brain and cognition Nat Rev Neurosci 2008 12 58 65 18094706 10.1038/nrn2298

[B9] Francis H Stevenson R The longer-term impacts of Western diet on human cognition and the brain Appetite 2013 12 119 128 23291218 10.1016/j.appet.2012.12.018

[B10] Crombie AP Ilich JZ Dutton GR Panton LB Abood DA The freshman weight gain phenomenon revisited Nutr Rev 2009 12 83 94 19178649 10.1111/j.1753-4887.2008.00143.x

[B11] Von Ah D Ebert S Ngamvitroj A Park N Kang DH Predictors of health behaviours in college students J Adv Nurs 2004 12 463 474 15533084 10.1111/j.1365-2648.2004.03229.x

[B12] Deliens T Clarys P Van Hecke L De Bourdeaudhuij I Deforche B Changes in weight and body composition during the first semester at university. A prospective explanatory study Appetite 2013 12 111 116 23402710 10.1016/j.appet.2013.01.024

[B13] Sigfusdottir ID Kristjansson AL Allegrante JP Health behaviour and academic achievement in Icelandic school children Health Educ Res 2007 12 70 80 16766605 10.1093/her/cyl044

[B14] Kristjansson AL Sigfusdottir ID Allegrante JP Helgason AR Adolescent Health Behavior, Contentment in School, and Academic Achievement Am J Health Behav 2009 12 69 79 18844522

[B15] Kristjansson AL Sigfusdottir ID Allegrante JP Health behavior and academic achievement among adolescents: the relative contribution of dietary habits, physical activity, body mass index, and self-esteem Health Educ Behav 2010 12 51 64 18541647 10.1177/1090198107313481

[B16] Keating XD Castelli D Ayers SF Strength exercise frequency, body mass index, and academic performance among university students J Strength Cond Res 2013 Published ahead-of-print 10.1519/JSC.0b013e318276bb4c 23096065

[B17] Anand V A study of time management: the correlation between video game usage and academic performance Cyberpsychol Behav 2007 12 552 559 17711364 10.1089/cpb.2007.9991

[B18] Aertgeerts B Buntinx F The relation between alcohol abuse or dependence and academic performance in first-year college students J Adolesc Health 2002 12 223 225 12225733 10.1016/s1054-139x(02)00362-2

[B19] Curcio G Ferrara M De Gennaro L Sleep loss, learning capacity and academic performance Sleep Med Rev 2006 12 323 337 16564189 10.1016/j.smrv.2005.11.001

[B20] Eliasson AH Lettieri CJ Early to bed, early to rise! Sleep habits and academic performance in college students Sleep Breath 2010 12 71 75 19603214 10.1007/s11325-009-0282-2

[B21] Richardson M Abraham C Bond R Psychological correlates of University Students’ Academic performance: a systematic review and meta-analysis Psychol Bull 2012 12 353 387 22352812 10.1037/a0026838

[B22] Singh A Uijtdewilligen L Twisk JWR van Mechelen W Chinapaw MJM Physical activity and performance at school a systematic review of the literature including a methodological quality assessment Arch Pediatr Adolesc Med 2012 12 49 55 22213750 10.1001/archpediatrics.2011.716

[B23] Neumark-Sztainer DR Wall MM Haines JI Story MT Sherwood NE van den Berg PA Shared risk and protective factors for overweight and disordered eating in adolescents Am J Prev Med 2007 12 359 369 17950400 10.1016/j.amepre.2007.07.031

[B24] Vereecken CA Maes L A Belgian study on the reliability and relative validity of the Health Behaviour in School-Aged Children food-frequency questionnaire Public Health Nutr 2003 12 581 588 14690039 10.1079/phn2003466

[B25] Steptoe A Wardle J The European health and behaviour survey: the development of an international study in health psychology Psychol Health 1996 12 49 73

[B26] Lefevre J Matton L Wijndaele K Duvigneaud N Thomis M Duquet W Philippaerts R Validity of the Flemish Physical Activity Computerized Questionnaire (FPACQ) Med Sci Sports Exerc 2006 12 S562 S562 10.1080/02701367.2007.10599427 17941534

[B27] Cohen S Kamarck T Mermelstein R A Global Measure of Perceived Stress J Health Soc Behav 1983 12 385 396 6668417

[B28] Severiens S ten Dam G Leaving college: a gender comparison in male and female-dominated programs Res High Educ 2012 12 453 470

[B29] Sheard M Hardiness commitment, gender, and age differentiate university academic performance Br J Educ Psychol 2009 12 189 204 18466672 10.1348/000709908X304406

[B30] Florin TA Shults J Stettler N Perception of overweight is associated with poor academic performance in US adolescents J Sch Health 2011 12 663 670 21972986 10.1111/j.1746-1561.2011.00642.x

[B31] Taras H Potts-Datema W Obesity and student performance at school J Sch Health 2005 12 291 295 16179079 10.1111/j.1746-1561.2005.00040.x

[B32] Florence MD Asbridge M Veugelers PJ Diet quality and academic performance J Sch Health 2008 12 209 215 18336680 10.1111/j.1746-1561.2008.00288.x

[B33] Chambel MJ Curral L Stress in academic life: work characteristics as predictors of student well-being and performance Applied Psychology: An International Review 2005 12 135 147

[B34] MacKinnon DP Fairchild AJ Fritz MS Mediation analysis Annu Rev Psychol 2007 12 593 614 16968208 10.1146/annurev.psych.58.110405.085542 PMC2819368

[B35] Duckworth AL Seligman MEP Self-discipline outdoes IQ in predicting academic performance of adolescents Psychol Sci 2005 12 939 944 16313657 10.1111/j.1467-9280.2005.01641.x

