# Correction: weight, socio-demographics, and health behaviour related correlates of academic performance in first year university students

**DOI:** 10.1186/1475-2891-13-16

**Published:** 2014-02-08

**Authors:** Tom Deliens, Peter Clarys, Ilse De Bourdeaudhuij, Benedicte Deforche

**Affiliations:** 1 Department of Human Biometry and Biomechanics, Vrije Universiteit Brussel, Brussels, Belgium; 2 Department of Movement and Sports Sciences, Ghent University, Ghent, Belgium

## Correction

After publication of this article [1], we noted an error in the values of the “Initial WC” row of Table 1. The correct values are presented here (Table 1).

**Table 1:** Descriptive statistics of possible influencing factors of GPA in first year university students (%, Mean ± SD, n = 101), subdivided into students who passed (n = 52), failed (n = 22) or did not attend all final course exams (n = 27)

**Measures**	**All n = 74**	**Passed n = 52**	**Failed n = 22**	**Did not attend all course exams n = 27**
***GPA (%)***	64.3 ± 9.2	68.3 ± 6.9	54.7 ± 6.2	/
***Demographics***				
Gender (% of females)	77.0	82.7	63.6	40.7
Age (yrs)	18.0 ± 0.6	17.9 ± 0.5	18.1 ± 0.8	18.3 ± 0.9
Ethnicity (% of students of which one of the parents is from foreign origin)	20.9	23.3	15.4	20.0
Residency (% living in student residence)	47.3	48.1	45.5	25.9
GPA in the last year of secondary school (%)	68.6 ± 7.5	70.0 ± 6.9	65.0 ± 8.0	63.6 ± 6.3
***Socio-Economic Status (SES)***^***c***^				
Education father (% diploma higher education)	57.2	48.3	77.0	46.7
Education mother (% diploma higher education)	69.0	70.0	66.6	60.0
***Smoking (% non-smokers)***	95.9	96.2	95.5	96.2
***Dieting status (% dieters)***^***b***^	11.0	9.8	13.6	11.5
***Anthropometrics***				
Initial weight (kg)	61.8 ± 9.3	61.0 ± 8.0	63.5 ± 12.0	66.7 ± 14.0
Initial BMI (kg/m^2^)	21.7 ± 2.7	21.5 ± 2.5	22.0 ± 3.1	22.1 ± 3.3
Initial fat% (%)	22.5 ± 7.1	22.8 ± 7.4	21.9 ± 6.7	19.2 ± 6.5
Initial WC (cm)	70.4 ± 6.4	69.6 ± 5.6	72.3 ± 7.9	73.5 ± 8.0
Weight change (kg)	0.7 ± 2.0	0.4 ± 1.9	1.6 ± 1.8	1.6 ± 2.2
BMI change (kg/m^2^)	0.3 ± 0.8	0.1 ± 0.8	0.5 ± 0.7	0.4 ± 0.8
Fat% change (%)	1.0 ± 2.4	0.7 ± 2.5	1.5 ± 2.0	0.5 ± 2.8
WC change (cm)	-0.0 ± 2.3	-0.5 ± 2.3	1.0 ± 2.1	1.2 ± 2.2
***Physical activity***^***d***^				
Active transportation (walking and cycling) (min/week)	179.7 ± 123.5	175.7 ± 119.5	189.5 ± 135.3	193.5 ± 118.4
Sport participation (min/week)	146.1 ± 180.1	152.7 ± 180.4	130.7 ± 182.9	117.4 ± 161.0
Total physical activity (min/week)	324.8 ± 211.7	326.8 ± 220.3	320.2 ± 195.4	310.8 ± 222.8
***Sedentary behaviour***^***a***^				
TV/DVD watching on weekdays (hours/day)	1.2 ± 0.8	1.2 ± 0.8	1.1 ± 0.8	1.2 ± 0.8
TV/DVD watching on weekend days (hours/day)	2.1 ± 1.1	2.1 ± 1.2	1.9 ± 1.0	2.2 ± 1.3
Reading and studying on weekdays (hours/day)	1.8 ± 1.1	1.8 ± 1.2	1.9 ± 0.8	1.9 ± 1.1
Reading and studying on weekend days (hours/day)	2.9 ± 1.5	2.8 ± 1.6	3.1 ± 1.5	2.8 ± 1.5
Computer activities on week days (hours/day)	1.7 ± 1.3	1.7 ± 1.4	1.7 ± 1.2	1.9 ± 1.2
Computer activities on weekend days (hours/day)	1.9 ± 1.2	1.8 ± 1.3	2.1 ± 1.0	2.3 ± 1.5
Video games on weekdays (hours/day)	0.2 ± 0.6	0.1 ± 0.4	0.4 ± 0.9	0.3 ± 1.0
Video games on weekend days (hours/day)	0.4 ± 1.0	0.3 ± 0.9	0.5 ± 1.2	0.6 ± 1.1
***Eating habits***				
Eating breakfast (#/week)^a^	5.7 ± 2.2	5.5 2.3	6.0 ± 2.0	5.8 ± 2.3
Eating lunch (#/week)^a^	6.6 ± 1.2	6.5 ± 1.3	6.6 ± 1.0	6.7 ± 0.9
Eating dinner (#/week)^a^	6.7 ± 0.9	6.7 ± 0.7	6.6 ± 1.3	6.8 ± 0.5
Eating at home with parents (#/week)^a^	3.8 ± 2.1	3.6 ± 2.1	4.1 ± 2.1	4.6 ± 2.1
Eating at student restaurant (#/week)^a^	1.2 ± 1.5	1.0 ± 1.1	1.8 ± 2.1	1.8 ± 1.9
Eating at fast food restaurant (#/week)^a^	0.3 ± 0.4	0.3 ± 0.4	0.3 ± 0.3	0.4 ± 0.3
Eating at other kind of restaurant (#/week)^a^	0.3 ± 0.3	0.3 ± 0.3	0.3 ± 0.3	0.4 ± 0.3
Eating at a friend’s place (#/week)^a^	0.4 ± 0.5	0.4 ± 0.4	0.4 ± 0.6	0.5 ± 0.5
Fruit consumption (#/day)^b^	1.0 ± 1.0	1.0 ± 1.1	0.9 ± 0.6	1.0 ± 1.1
Vegetable consumption (#/day)^b^	1.2 ± 0.7	1.2 ± 0.7	1.2 ± 0.6	1.3 ± 1.0
Soda consumption (#/day)^b^	0.8 ± 1.1	0.6 ± 0.9	1.2 ± 1.3	1.2 ± 1.3
French fries consumption (#/week)^b^	0.1 ± 0.1	0.1 ± 0.1	0.1 ± 0.1	0.1 ± 0.1
Fast food consumption (#/week)^b^	0.7 ± 0.9	0.7 ± 0.9	0.8 ± 0.9	0.9 ± 0.9
***Alcohol***				
Frequency of alcohol use (#/week)^b^	0.8 ± 1.5	0.6 ± 1.0	1.3 ± 2.4	0.8 ± 1.3
Frequency of alcohol consumptions (# on drinking days)^c^	2.7 ± 2.0	2.6 ± 1.9	2.9 ± 2.2	3.1 ± 3.0
***Sleeping habits***^***c***^				
Hours of sleep on weekdays (hours/day)	7.8 ± 1.0	7.8 ± 1.0	7.9 ± 1.1	7.6 ± 0.9
Hours of sleep on weekend days (hours/day)	9.4 ± 1.2	9.4 ± 1.2	9.3 ± 1.3	9.2 ± 1.2
***Stress***				
Mental stress (PSS score*)^e^	13.6 ± 5.9	13.5 ± 6.0	13.8 ± 5.8	14.4 ± 6.5
